# *Caenorhabditis elegans* as an In Vivo Model for the Discovery and Development of Natural Plant-Based Antimicrobial Compounds

**DOI:** 10.3390/ph16081070

**Published:** 2023-07-27

**Authors:** Samah H. O. Zarroug, Juhaina S. Bajaman, Fatheia N. Hamza, Rimah A. Saleem, Hana K. Abdalla

**Affiliations:** 1 Department of Pharmacology, College of Medicine, Alfaisal University, Takassusy Road, Riyadh 11533, Saudi Arabia; 2 Department of Biochemistry, College of Medicine, Alfaisal University, Takassusy Road, Riyadh 11533, Saudi Arabia; fahamza@alfaisal.edu (F.N.H.); rsaleem@alfaisal.edu (R.A.S.); 3 Department of Microbiology, College of Medicine, Alfaisal University, Takassusy Road, Riyadh 11533, Saudi Arabia; haabdalla@alfaisal.edu

**Keywords:** multidrug-resistant pathogens, *C. elegans*, natural products, antimicrobial activity

## Abstract

Antimicrobial resistance (AMR) due to the prevalence of multidrug-resistant (MDR) pathogens is rapidly increasing worldwide, and the identification of new antimicrobial agents with innovative mechanisms of action is urgently required. Medicinal plants that have been utilised for centuries with minor side effects may hold great promise as sources of effective antimicrobial products. The free-living nematode *Caenorhabditis elegans (C. elegans)* is an excellent live infection model for the discovery and development of new antimicrobial compounds. However, while *C. elegans* has widely been utilised to explore the effectiveness and toxicity of synthetic antibiotics, it has not been used to a comparable extent for the analysis of natural products. By screening the PubMed database, we identified articles reporting the use of the *C. elegans* model for the identification of natural products endowed with antibacterial and antifungal potential, and we critically analysed their results. The studies discussed here provide important information regarding “in vivo” antimicrobial effectiveness and toxicity of natural products, as evaluated prior to testing in conventional vertebrate models, thereby supporting the relevance of *C. elegans* as a highly proficient model for their identification and functional assessment. However, their critical evaluation also underlines that the characterisation of active phytochemicals and of their chemical structure, and the unravelling of their mechanisms of action represent decisive challenges for future research in this area.

## 1. Introduction

Antimicrobial resistance (AMR) occurs when pathogens such as bacteria, fungi, viruses, and parasites evolve in ways rendering current treatments, including antibiotics, ineffective. Despite efforts and initiatives promoted by the World Health Organisation (WHO), the spiralling increase in AMR remains one of the top 10 health issues worldwide [1,2,3]. While the overprescription and misapplication of antimicrobials represent major factors underlying the emergence of multidrug-resistant (MDR) pathogens [4], worldwide trade, migration, and travel have accelerated their spread [5].

A recent study reported that while globally, in 2019, 4.95 million deaths could be considered as associated with AMR, 1.27 million of them could be directly attributed to infections by MDR pathogens [6]. If no action is taken, the WHO has warned that this figure could reach 10 million deaths per year by 2050 [6], thus surpassing cancer, diabetes, and heart disease as the leading cause of death worldwide [7]. Moreover, the unavailability of effective therapies, leading to prolonged hospital stays and work absenteeism, could increasingly burden health systems’ economies due to higher treatment costs [4].

In 2019, three infectious diseases were associated with AMR [6] worldwide. They include lower respiratory infections, linked to >1.5 million deaths, followed by bloodstream and intra-abdominal infections [6]. Importantly, six pathogens, namely, *Escherichia coli, Staphylococcus aureus, Klebsiella pneumoniae*, *Streptococcus pneumoniae*, *Acinetobacter baumannii*, and *Pseudomonas aeruginosa*, were responsible for a total of 929,000 deaths attributable to AMR worldwide [6], with *E coli* being the leading cause [6]. In particular, *Enterococcus faecium*, *Staphylococcus aureus*, *Klebsiella pneumoniae*, *Acinetobacter baumannii*, *Pseudomonas aeruginosa*, and *Enterobacter* species, collectively defined as ESKAPE pathogens, have developed resistance to several classes of antibiotics, allowing them to “escape” the bacteriostatic or bactericidal effects of current treatments [8]. These pathogens represent leading causes of nosocomial infections and are associated with high mortality rates, prolonged hospital stays, and increased healthcare costs [9,10,11,12]. New antimicrobials targeting ESKAPE and other pathogens are urgently needed [13].

*Caenorhabditis elegans* (*C. elegans*) has widely been used in the past two decades as a model organism for drug discovery [14,15] and to explore “in vivo” mechanisms of action of previously identified compounds. Most importantly, it has also been proficiently utilised for the identification of novel antimicrobials, with a major focus on libraries of synthetic compounds [16,17].

Plant-based natural products have been used for centuries as sources of antimicrobial compounds, and they may provide safe, alternative antimicrobial treatments. Considering the role of *C. elegans* in the discovery of novel drugs and in the unravelling of their molecular mechanisms, here we highlight the importance of using this model in the study of plant-derived antibacterial and antifungal compounds and review previous studies in this research area.

## 2. Current Issues with Antimicrobial Drug Discovery

Current antimicrobial drug discovery rates fail to meet the urgent demand for novel compounds to treat life-threatening infections caused by MDR pathogens. Limited returns on investment and scarce funding are some of the factors contributing to insufficient antimicrobial drug development, which requires approximately 10 years from preclinical trials until market authorisation according to WHO estimations [2]. Indeed, between 2020 and 2021, only one antimicrobial compound has been approved by the US Food and Drug Administration (FDA) and the European Medicines Agency (EMA) [18]. Moreover, compounds lacking cross-resistance, with novel mechanisms of action or molecular targets, or those belonging to new classes of drugs are relatively rare. In 2022, WHO reported that out of 32 prioritised new antimicrobial compounds in clinical trials, only 6 could be considered innovative [2].

Initial stages of antimicrobial drug screening typically utilise in vitro methods focusing on direct bacteriostatic or bactericidal effects. However, the use of these methods in the screening of compounds with novel mechanisms of action presents several limitations. Culturing of the defined microorganisms in viable conditions may be difficult. The high concentrations of compounds used for in vitro screening may prove toxic or may have poor pharmacokinetic properties in later stages of development [19,20]. Moreover, some compounds are unable to cross the MDR-barrier of Gram-negative bacteria [21] or are ineffective when tested in vivo [19,22,23].

Overall, current in vitro assays appear to be inadequate for the discovery of novel antimicrobials [24], and more robust in vivo multicellular models are necessary.

## 3. Advantages of *C. elegans* as a Model for the Screening of Novel Compounds

To address these issues, in the last decade, antimicrobial drug discovery technology has moved towards the use of whole animal models, such as zebrafish and *C. elegans,* for the screening of new compounds and the identification of their mechanisms of action.

*C. elegans*, a bacteria-eating organism, can be infected and killed by a variety of human pathogens, including Gram-negative bacteria such as *Pseudomonas aeruginosa* and *Salmonella enterica*; Gram-positive bacteria such as *Staphylococcus aureus, Streptococcus pneumoniae,* and *Enterococcus faecalis*; and fungi such as those of the *Candida* species [16,17,24,25].

**Figure 1:**
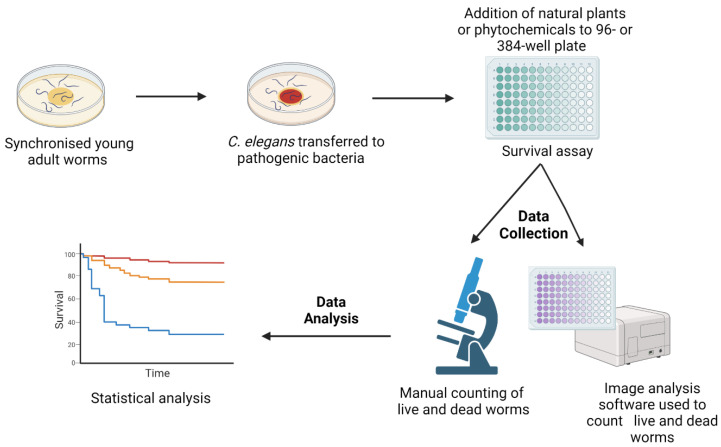
A schematic diagram of the survival assays used for the *C. elegans*-based screening of novel compounds with antimicrobial activity. Synchronised *C. elegans* are generated either by egg laying or the bleaching method. Worms are grown in *E. coli* OP50 plates for 2–3 days at 20 °C until their adulthood. Subsequently, they are transferred to plates seeded with pathogenic microbes. Following an incubation period, worms are transferred to 12-, 24-, 96-, or 384-well assay plates containing different concentrations of antimicrobial compounds. Numbers of live and dead worms can be scored manually under the microscope or by imaging software using a cell permeable dye identifying dead nematodes. The image was created with BioRender.com.

To screen for novel antimicrobial compounds, the *C. elegans* natural food source, *E. coli* OP50, is replaced by a pathogenic bacterium, e.g., *Pseudomonas aeruginosa*. Survival assays are performed either on agar or in liquid media [16,25,26,27]. Initially, worms are incubated with pathogenic organisms to establish infection before transferring them into assay plates (12-, 24-, 96-, or 384-well plates) that are subsequently added with different concentrations of novel compounds. The number of live and dead worms can be counted visually under the microscope or, in automated assays, by using a cell-permeable dye, identifying dead worms, and image analysis software [17,28] (Figure 1). Notably, automated assays with 10–20 worms per well in 96- or 384-well plates allow for the screening of large numbers of compounds [29].

The main advantage of the live animal model is that it not only allows for the assessment of direct antimicrobial effects of defined agents with novel mechanisms of action or molecular targets, but it also addresses their potential role as immune modulators [30], indirectly contributing to fighting microbial virulence and increasing the likelihood of host survival. Moreover, the use of a whole live animal model provides valuable information on systemic toxicity of specific compounds in the early stages of drug discovery.

Importantly, the small size and short lifespan of *C. elegans* convincingly support its suitability as a model organism. Indeed, *C. elegans* is a 1 mm long, free-living, transparent nematode that develops from a hatched egg to adulthood in 3 days at 20 °C. Once they reach adulthood, wild-type worms live for three additional weeks. The short lifespan and quick generation time accelerate the antimicrobial discovery process and critically favour its automation.

*C. elegans* was the first multicellular organism whose genome was fully sequenced (*C. elegans* Sequencing Consortium 1998). Most notably, *C. elegans* is a self-fertilised organism, and all offspring are genetically identical, thus providing a distinct advantage compared to fruit flies and murine models [31]. Indeed, inbreeding of mutant strains to clarify mechanisms of action of novel compounds can be easily performed starting from a highly homogeneous genetic background.

Thus, *C. elegans* qualifies as an excellent model organism, bridging the gap between in vitro assays and the complexities of vertebrate models.

## 4. Natural Plants as Sources of Novel Antimicrobial Agents

Natural plants have long been used in traditional medicine [32,33]. In particular, many plant species have been utilised to treat microbial infections, and scientists have capitalised on traditional knowledge to screen natural products with potential antimicrobial activity and to attempt to decipher their mechanisms of action [32].

The extraction of novel antimicrobial agents from natural plants is cost-effective compared to synthetic chemistry methods, since plants are usually easily available and purification processes are relatively inexpensive [33]. This is particularly important in developing countries where a variety of infectious diseases are endemic. Natural plant extracts may provide a more accessible and affordable treatment for some of them.

Notably, natural plants offer a range of secondary metabolites, including phenols, flavonoids, sulphur compounds, phenolic glycosides, saponins, unsaturated lactones, cryogenics, and/or glucosinolates, thereby representing rich sources of novel compounds with antimicrobial properties [34,35,36]. These metabolites are usually not necessary for plant survival but can offer protection against predators and pathogens [37]. Indeed, many of them have already been shown to display antimicrobial activity [34,38,39]. Their diversity suggests that a large number of plant species can be explored as potential sources of antimicrobial activity [37,39,40,41].

Importantly, antimicrobial compounds extracted from natural plants have been reported to be characterised by fewer side effects compared to synthetic agents [42,43], possibly due to the complex mixture of different metabolites present in natural plants, which may have synergistic effects or counteract the toxicity of individual components. While this complexity could hinder the standardisation of natural plant extracts, it provides a strong incentive for an updated analysis of interactions occurring between different metabolites, also addressing their antioxidant, anti-inflammatory, and anticancer properties [44,45].

## 5. Methodology

All previous works on the use of *Caenorhabditis elegans* as an in vivo model for the discovery and development of natural plant-based antimicrobial compounds were collected from the PubMed database, examined, and reported in the present review. A total of 203 peer-reviewed papers published in the English language were included in this review. The following search terms were used: “Natural products, *Caenorhabditis elegans*, antibacterial activity”, overall yielding 41 entries; and “Natural products, *Caenorhabditis elegans*, antifungal activity”, overall yielding 10 entries. One entry shared both terms. Reports published until April 2023 were considered. Entries focusing on anthelminthic activity were excluded. Within this database, our analysis focused on a critical evaluation of natural products active against MDR pathogens and tested in the *C. elegans* model.

## 6. Natural Products Active against Bacterial Infection

In vitro and in vivo antibacterial activities of natural plant products, as tested using the *C. elegans* model, are illustrated in Table 1.

*Staphylococcus aureus* (*S. aureus*), a Gram-positive bacterium, frequently implicated in various community- and hospital-acquired infections, has developed resistance against penicillin and semisynthetic methicillin. The ability to produce potent toxins and enzymes and form biofilms makes these bacteria highly feared pathogens. Methicillin-resistant *S. aureus* (MRSA) has become widespread in all settings, and new strains continue to emerge and develop higher resistance to the few available antibiotics [75].

A liquid-based assay used to screen novel compounds has shown that natural extracts from *Nypa fruticans* (mangrove palm), *Swietenia macrophylla* (big leaf mahogany), *Curcuma longa* (turmeric), *Eurycoma longifolia* (longjack), *Orthosiphon stamineus* (cat’s whiskers), and *Silybum eburneum* (milk thistle) increase the lifespan of *S. aureus*-infected *C. elegans* by 2.8-fold [46]. *N. fruticans* husks and *C. longa* inhibit *S. aureus* growth at concentrations <200 μg/mL. Furthermore, extracts from *N. fruticans* roots, *S. macrophylla* seeds, and *O. stamineus* leaves protect worms from MRSA, and reduced colonisation of the nematode intestines by *S. aureus* was observed following treatment with *N. fruticans* root and *O. stamineus* leaf extracts. These results suggest that products from these plants, historically used for their analgesic, anti-inflammatory, and antioxidative effects in Southeast Asia, could promote host immunity and/or decrease bacterial virulence [46].

In another study, a herbal formulation used to treat wound infections and derived mainly from leaves and bark of plants such as *Azadirachta indica* (Neem), *Acacia nilotica* (Babul), *Ocimum sanctum* (Tulsi), and *Annona squamosa* (sugar apple) showed promising activity upon repeated exposure of *C. elegans* to *S. aureus*. This formulation, containing the *Curcuma longa* (turmeric) rhizome and seed oil of *Ricinus communis* (castor oil), inhibits bacterial growth and production of staphyloxanthin, a quorum-sensing (QS)-regulated pigment, at concentrations ≥0.025% *v*/*v*. In addition, it prevents biofilm formation and significantly modifies the bacterial transcriptome by targeting genes associated with haemolysis, virulence, enzyme activity, basic cellular processes, quorum-sensing, and transcriptional regulation [51].

*P. aeruginosa* is another opportunistically resistant bacterium posing major healthcare challenges, due to a range of virulence factors [76], with intrinsic and acquired mechanisms including low outer membrane permeability, multidrug efflux pumps, and enzymes. Moreover, *P. aeruginosa* demonstrates adaptive resistance through persistent biofilm formation in infected tissues [77].

Secondary metabolites from endophytic fungi of *Alangium*, *Angelica sinensis* (female ginseng), *Bupleurum chinense*, *Herba plantaginis* (plantain), *Schisandra chinensis* (magnolia vine fruit), *Herba menthae* (mint), and *Stephania japonica* (snake vine), used in traditional Chinese medicine (TCM), were tested in a liquid-based, slow-killing assay with *C. elegans* infected with MDR *P. aeruginosa.* This study identified 36 extracts prolonging *C. elegans* survival, and 4 with antimicrobial activity against *P. aeruginosa*. The 18s rRNA amplicon identified the fungal strains producing these four extracts with in vitro and in vivo activity as *Alternaria sp*. from *Angelica sinensis*; *Herba plantaginis* and *Phoma exigua* from *Alangium sp*.; and *Aspergillus sydowii* from *Plantago depressa* [54].

In another study, polysaccharide extracts from *Sophora moorcroftiana* seeds, a medicinal shrub endemic to China, were shown to improve the lifespan of *C. elegans* as well as its reproductive capacity upon *P. aeruginosa* infection, and they demonstrated specific antibacterial activity [55]. Both studies suggest that these natural compounds may enhance host immunity and/or reduce bacterial virulence.

*Swietenia macrophylla* (*S. macrophylla*), a source of limonoids (modified terpenoids), has shown antimicrobial activity against MRSA [46]. Moreover, *S. macrophylla* seed extracts significantly improve the survival of *P. aeruginosa*-infected *C. elegans*. Notably, seed ethyl acetate and methanol extracts do not inhibit *P. aeruginosa* growth. However, *S. macrophylla* enhances the expression of the immune modulator gene *lys-7*, which is known to be suppressed by the QS mechanisms of *P. aeruginosa* [56,57].

Modulation of the QS mechanisms by natural products is further supported by studies highlighting the antibacterial effects of CBO. In vitro studies have shown that CBO inhibits elastases A and B and regulates signalling molecules of *P. aeruginosa*. Importantly, it also decreases the expression and production of other virulence factors, such as chitinase, pyocyanin, exopolysaccharide, and biofilm formation, at subinhibitory concentrations (<3.2%). Treatment of *P. aeruginosa*-infected *C. elegans* with 1.6% *v/v* clove oil improves worm survival in vivo [58,59]. Similar effects were observed by using *Murraya koenigii* (curry tree), a medicinal plant with anti-infective and antioxidant properties [60].

Marine plants, such as the macroalgae *Chondrus crispus (C. crispus)*, a red seaweed, are rich sources of compounds with immunomodulatory abilities [78]. A water extract of *C. crispus* has been shown to improve the survival of *P. aeruginosa*-infected *C. elegans* at a 500 µg/mL concentration. These effects result from both host immune-system activation through the *pmk-1*, *daf-2*/*daf-16*, and *skn-1* pathways, and QS and virulence gene suppression [61]. Furthermore, an extract of *Ascophyllum nodosum*, a brown seaweed, promotes the survival of *C. elegans* through similar mechanisms and by significantly reducing biofilm formation by *P. aeruginosa* [62].

Ayurveda, an ancient holistic approach used for disease prevention and treatment, has also been extensively studied in the context of innovative drug discovery. For instance, TF, a formulation of the three plants *Phyllanthus emblica* (Indian gooseberry), *Terminalia bellerica* (Bibhitaka), and *Terminalia chebula* (Chebulic Myrobalan), is commonly used in Ayurvedic medicine to improve general health and to treat a variety of diseases [79].

*C. elegans* has been used as an in vivo model to test TF antibacterial activity against *S. aureus*, *P. aeruginosa*, *S. pyogenes*, *S. marcescens*, and *C. violaceum* [63]. All pathogens except *S. pyogenes* showed reduced virulence (18–45%) towards the nematode host following pretreatment with TF. Moreover, *C. elegans* pretreated with TF also showed an increased survival rate (14–41%) following infection with these five pathogens. In addition, TF is effective in worms infected with *S. marcescens*, *C. violaceum*, or *S. aureus*. However, TF is ineffective in worms exposed to a mixed population of *S. aureus* and *P. aeruginosa*. TF antibacterial activity can be attributed to a combination of reduced QS pigment production in Gram-negative bacteria, virulent enzyme inhibition in *S. aureus*, and upregulation of the host immune defence accompanied by decreased biofilm formation by *S. marcescens* and *S. aureus*. In a similar study, bacteria pretreated with TF leaf extracts showed decreased virulence associated with QS modifications [79].

*Psoralea corylifolia* (Babchi), a herb used in traditional Chinese medicine and Ayurveda to treat skin diseases [80], has also been studied for its QS-dampening constituents. Its seed extracts contain bakuchiol, a terpenophenol that inhibits in vitro biofilm formation by *P. aeruginosa*, *A. hydrophila*, *C. violaceum*, *S. marcescens*, and *L. monocytogenes*.

To study their effects in vivo, *C. elegans* infected with *P. aeruginosa* were treated with 1000 µg/mL *P. corylifolia* seed extract. Worms survived longer than untreated nematodes [64]. While increased survival was also observed in a similar study testing *Mangifera indica* (mango) leaf extracts [65], underlying in vivo mechanisms remain to be investigated.

*Streptococcus pyogenes (S. pyogenes*), a Gram-positive, β-haemolytic streptococcus, can cause highly contagious infections with serious complications, such as rheumatic fever. Although first-line treatment with penicillin is still effective, some resistant strains are emerging [81]. The bioflavonoid fukugiside from *Garcinia travancorica* (mangosteen) has shown promising concentration-dependent antibacterial properties against *S. pyogenes* in vitro by downregulating various virulence genes and reducing its ability to evade phagocytosis. Fukugiside significantly improves the survival rate of *C. elegans* infected with *S. pyogenes* without displaying any toxicity [66]. Betulin, a triterpenoid present in the bark of birch trees, has also shown similar effects in vivo [82]. Interestingly, in an innovative antimicrobial approach, extracts of *Tripterygium wilfordii* (Thunder God Vine) were able to photosensitise *S. pyogenes* and *S. aureus* for the treatment of skin infections. Accordingly, the combination of *T. wilfordii* extract and antimicrobial photodynamic therapy improved the lifespan of *C. elegans* infected with *S. pyogenes* [67].

Among pathogens that conventional antimicrobials cannot eradicate [83], shigatoxin-producing enterohemorrhagic *E coli* 0157:H7 causes bloody diarrhoea, which can only be managed supportively. Within this context, the *C. elegans* model was used to test the antibacterial properties of clove oil, known to downregulate various virulence factors, including QS molecules, and biofilm formation [58,59]. Indeed, eugenol, the major constituent of clove oil, displays high efficacy against *E coli* 0157:H7 and prolongs nematode survival [68].

Another gut pathogen, *Salmonella typhimurium*, is susceptible to *B. oleracea var. Botrytis* (cauliflower) byproduct extracts. Studies in cauliflower-treated *C. elegans* have shown a dose-dependent reduction in *Salmonella typhimurium* virulence factors, significantly reduced colonisation, and prolonged nematode survival [69,70].

In addition to flavonoids, phytoestrogens such as honokiol and magnolol, derived from magnolia species, have demonstrated antibacterial activity [71]. The survival of *Vibrio cholerae*-infected *C. elegans* can be significantly prolonged by treatment with these polyphenols. In vitro studies suggest that these compounds decrease host inflammatory responses to this potent diarrhoeal pathogen [74].

*Burkholderia pseudomallei*, a tropical pathogen that causes serious melioidosis, shows reduced pathogenicity in vivo when infected *C. elegans* are treated with curcumin (turmeric). Further investigations have shown that this effect relies on alterations of the bacterial transcriptome, resulting in the attenuation of virulence factors [72].

## 7. Natural Products Active against Fungal Infection

The *C. elegans* model has also successfully been used for the discovery of antifungal natural products (Table 2).

*Candida albicans (C. albicans)*, causing opportunistic infections, has represented a major target for the discovery of natural antifungal products. The limited array of antifungal drugs and the intrinsic ability of *C. albicans* to quickly develop tolerance highlight the significance of the *C. elegans* infection model in this regard [89].

A total of 2560 natural products were screened to identify compounds favouring the survival of *C. albicans*-infected nematodes, and 12 triterpenoid-based saponins significantly prolonged it. Six of these compounds also inhibited in vitro growth of the yeast, including its resistant strains, and two prevented biofilm formation [87].

Saponins are plant glycosides known in traditional medicine for their antitumorigenic, antimicrobial, and anti-inflammatory properties. Although they can be toxic at high concentrations due to their haemolytic properties [90], they have shown promising potential as enhancers of photosensitiser uptake in combination with photodynamic treatments [87].

Gallic acid and gallates are secondary metabolites present in various plants and are known for their antioxidant and anti-inflammatory properties [91]. In vitro studies support their antifungal effects against *Candida* spp., *Cryptococcus* spp., *Paracoccidioides* spp., and *Histoplasma capsulatum* [92]. The in vivo model of *C. albicans*–*C. elegans* indicates that they significantly increase the nematode lifespan at >1 µg/mL concentrations, although their toxicity is also increased at high concentrations [84].

Another hydrophobic polyphenol, thymol, also prolongs the survival of *C. albicans*-infected worms and shows antibiofilm activity in vitro. This compound, extracted from thyme, also prevents dysregulation of the host p38 MAPK signalling pathway, playing a key role in the immune response [93].

An investigative study identified 15 out of 1266 tested compounds that promoted the longevity of infected worms and prevented the fungal yeast–hyphal transition. They included plant-based compounds, caffeic acid phenethyl ester (CAPE), and lapachol [85]. CAPE is a polyphenolic ester naturally occurring in plants, and its bioactive form can be extracted from honeybee hive propolis. Its mechanism of action has been proposed to rely on yeast gene suppression and immunomodulation [94]. On the other hand, lapachol, a naphthoquinone derived from the bark of *Tabebuia avellaneda* (lapacho tree), is used in traditional Amazonian medicine as an antibacterial and antiparasitic agent [45]. Both compounds were shown to inhibit *C. albicans* biofilm formation in vitro, although only CAPE demonstrated antifungal effects in a murine model [85].

Fungal filamentation and biofilm formation are key virulence factors accounting for *C. albicans* pathogenicity by allowing microbes to colonise mucosal surfaces and medical devices, and to invade human tissues. Moreover, the biofilm matrix renders fungi resistant to immune defences and drugs, leading to intractable infections [95]. The non-toxic naturally occurring antifungals magnolol and honokiol significantly suppress in vitro yeast cell adhesion, hyphal growth, and biofilm formation at 4–32 μg/mL concentrations by downregulating virulence genes, including those of the Ras1-cAMP-Efg1 pathway. Importantly, both honokiol and magnolol significantly prolong the lifespan of *C. albicans*-infected worms by preventing colonisation [86]. In a similar study, tetrandrine (TET), an alkaloid obtained from the roots of *Stephania tetrandra*, a medicinal herb known as Fang Ji used in Chinese herbal medicine for joint diseases, inhibited the yeast-to-hyphae transition and biofilm formation in vitro by downregulating the expression of genes from the Ras/cAMP pathway, responsible for QS and biofilm formation [96]. Accordingly, TET enhanced the lifespan of infected nematodes at the same concentration range as magnolol and honokiol [88]. 

Major issues related to the use of antifungal agents are associated with their adverse effects and drug-resistance development, since treatment requires long-term administration due to frequent relapses [97]. Therefore, natural plants may represent a safe and effective alternative and *C. elegans* may provide an early detection tool to identify compounds with selective toxicity against fungi and/or unravel alternative mechanisms of action inhibiting fungal infection.

## 8. Conclusions

Microbial diseases have long been treated with plant extracts. The growing issue of antibiotic resistance has prompted research into natural substances as cutting-edge antimicrobial agents. The discovery of natural substances with antibacterial and antifungal characteristics has greatly benefited from using *C. elegans* as an in vivo model. The *C. elegans* model has been used to investigate simultaneously the toxicity and antimicrobial efficacy of plant-based products using survival assays. Furthermore, the interactions between plant-based products and host–pathogen interactions have been studied to delineate the mechanisms of action involved, such as quorum-sensing inhibition and host immune-induction activity. Moreover, *C. elegans* mutant strains and molecular techniques have been utilised to decipher the signalling pathways involved in the antimicrobial efficacy of plant-based products. However, issues such as poorly standardised plant extract preparations and inadequate data on safety and toxicity still exist.

Most importantly, as clearly emerging from our study, for a large number of natural products, mechanisms of action are still poorly defined. Moreover, the nature and chemical structure of active compounds and secondary metabolites are frequently unclear. Addressing these issues will represent a major research challenge in the near future, and the use of the *C. elegans* model may powerfully contribute to this effort by accelerating the identification of effective phytochemicals and by more reliably delineating their mechanisms of action.

On the other hand, since converting plant products into innovative antimicrobial drugs requires major funding to pinpoint active ingredients and standardise production and distribution processes, the use of *C. elegans* may lessen the financial risk involved and help promote investment in this area.

## Figures and Tables

**Table 1:** Antibacterial activity of natural plant products in the *C. elegans* in vivo model. * MoA: mechanism of action; ** N.D.: not determined.

Active Compounds	Plant	Microorganism Tested	In Vitro	In Vivo (*C. elegans* Infection Model)	MoA *	References
			(MIC)	(EC_50_)		
Eupatorin, sinensetin, 3′-hydroxy-5, 6, 7,4′-tetramethox-yflavone, and rosmarinic acid	*Nypa fruticans* *Swietenia macrophylla* *Curcuma longa* *Eurycoma longifolia* *Orthosiphon stamineus* *Silybum eburneum*	*S. aureus* NCTC8325-4 and MRSA ATCC33591	<100 μg/mL	Reduced bacterial colonisation of the nematode intestines by *S. aureus* NCTC8325-4 and increased survival by 2.8-fold. Protected worms from MRSA ATCC33591 infection	Immune genes activation and inhibition of bacterial virulence	[46,47,48]
			No data			
			<125 μg/mL			
			N.D. **			
			N.D. **			
			100 μg/mL			
N.D. **	*Diplocyclos palmatus*	*S. marcescens PS1*	Inhibition of prodigiosin pigment production in *S. marcescens* at 2 mg/mL by 85%	Increased survival time by 50% Reduced colonisation of *S. marcescens*	Inhibition of QS-regulated biofilm formation and virulence factor production in *S. marcescens.* Upregulation of *daf-16* and immune-related genes (*clec-87*, *lys-7*, *bec-1*, and *clec-60*)	[49]
N.D. **	*Bifidobacterium* spp.	Enterohemorrhagic *Escherichia coli* (EHEC) O157:H7	98-fold reduction in AI-2 activity in EHEC O157:H7	Increased survival rate against EHEC O157:H7	Inhibition of virulence factor production in EHEC O157:H7	[50]
N.D. **	*Azadirachta indica*	*S. aureus*	0.025–0.5% *v*/*v*	Inhibition of bacterial growth and production of staphyloxanthin, a quorum sensing (QS)-regulated pigment, at concentrations ≥0.025% *v*/*v*	QS-mediated bacterial virulence and transcription of the genes encoding virulence factors	[51]
	*Acacia nilotica*					
	*Ocimum sanctum*					
	*Annona squamosa*					
	*Curcuma longa*					
	*Ricinus communis*					
N.D. **	Broccoli extract	*Escherichia coli O157:H7*	N.D. **	Increased survival rate of worms by 28.5%	Inhibition of QS-mediated bacterial virulence genes	[52]
Honokiol and magnolol	*Magnolia* sp.	Methicillin-resistant *Staphylococcus aureus (*MRSA*)*	N.D. **	Protected worms from MRSA infection and increased survival rate	Increased immune-modulatory effects via IFN-β and IFN-λ	[53]
N.D. **	*Angelica sinensis*	*P. aeruginosa*	In vitro inhibition of MDR*P. aeruginosa*	Prolonged *C. elegans* survival against MDR *P. aeruginosa*	N.D. **	[54]
	*Bupleurum chinense*					
	*Herba plantaginis*					
	*Herba menthae*					
	*Stephania japonica*					
	*Phoma exigua*					
	*Aspergillus sydowii*					
	*Herba plantaginis*					
	*Plantago depressa*					
N.D. **	*Sophora moorcroftiana*	*P. aeruginosa*	N.D. **	Improved *C. elegans* lifespan and reproductive capacity	N.D. **	[55]
Limonoids	*Swietenia macrophylla*	*P. aeruginosa* PA14	No inhibition	Prolonged *C. elegans* lifespan by reducing virulence factors and enhancing immune response	Activation of immune modulators gene *lys-7*, which suppresses QS-mediated mechanisms	[56,57]
N.D. **	Clove bud oil (CBO)	*P. aeruginosa*	CBO inhibited elastases A and B and regulated *P. aeruginosa* signalling molecules at concentrations (<3.2%)	Treatment of *P. aeruginosa*-infected *C. elegans* with <2% *v/v* clove oil improved worm survival in vivo	Immune genes activation, QS regulation	[58,59]
N.D. **	*Murraya koenigii*	*P. aeruginosa*	N.D. **	Improved the survival of infected worms	Immune genes activation	[60]
N.D. **	*Chondrus crispus*	*P. aeruginosa*	N.D. **	Improved survival of *P. aeruginosa*-infected *C. elegans* at a 500 µg/mL concentration	Immune genes activation (the *pmk-1*, *daf-2*/*daf-16*, and *skn-1*)	[61]
N.D. **	*Ascophyllum nodosum*	*P. aeruginosa*	N.D. **	Promoted the survival of *C. elegans* by significantly reducing biofilm formation by *P. aeruginosa*	Immune genes activation	[62]
Triphala (TF)	*Phyllanthus emblica*	*S. aureus, P. aeruginosa, S. marcescens,* and *C. violaceum*	N.D. **	Reduced virulence (18–45%) towards the nematode host and increase survival of *C. elegans* by 14–41%	N.D. **	[63]
	*Terminalia bellerica*					
	*Terminalia chebula*					
N.D. **	*Psoralea corylifolia*	*P. aeruginosa,*	Inhibited in vitro biofilm formation	1000 µg/mL *P. coryliflia* seed extract increased infected worms’ survival	Inhibition of QS-mediated virulence and biofilm (*LasR* and *RhlR* genes)	[64]
		*A. hydrophila,*				
		*C. violaceum,*				
		*S. marcescens,* and				
		*L. monocytogenes*				
N.D. **	*Mangifera indica*	*Chromobacterium violaceum (12472)* *Pseudamonas aeruginosa PAO1* *Aeromonas hydrophila WAF38*	*Chromobacterium violaceum (12472)*	*P. aeruginosa*-infected worms treated with *Mangifera indica* survived longer (72%) compared to untreated nematodes	N.D. **	[65]
			= 1000 μg/mL,			
			*P. aeruginosa* PAO1 and A. *hydrophila WAF38* = 2000 μg/mL			
Fukugiside	*Garcinia travancorica*	*Streptococcus pyogenes SF370*	60, 80, 100, and 120 µg/mL shows 70–90% biofilm inhibition	80 µg/mL concentration increased survival of *C. elegans* infected with *Streptococcus pyogenes*	Inhibition of virulence genes and phagocytic enhancement	[66]
N.D. **	*Tripterygium wilfordii*	*S. pyogenes* *S. aureus*	70–96% biofilm inhibition at 80 μg/mL	Improved the lifespan of *C. elegans* infected with *S. pyogenes*	Photosensitisation	[67]
			Photosensitise *S. pyogenes* and *S. aureus* for the therapy of skin infections			
Eugenol	Bay and clove essential oils	Enterohemorrhagic *Escherichia coli O157:H7 (EHEC)*	0.0005% inhibited 87% of EHEC biofilm. A 100% inhibition was reported at concentration of 0.005%	0.005% concentration prolonged *C. elegans* survival in the presence of EHEC	Biofilm inhibition	[68]
N.D. **	*B. oleracea var Botrytis* (cauliflower)	*Salmonella typhimurium CECT 443*	3% cauliflower	3% cauliflower prolonged *C. elegans* infected with *Salmonella typhimurium* and	Reduced virulence factors	[69,70]
				dose-dependently reduced *Salmonella typhimurium* virulence factors		
Honokiol and Magnolol	*Magnolia* sp.	*Vibrio cholerae*	N.D. **	Worm survival significantly prolonged and decreased host inflammatory responses	N.D.**	[71]
Curcumin	*Curcuma longa*	*Burkholderia pseudomallei*	N.D. **	Reduced pathogenicity and attenuation of virulence factors in *Burkholderia pseudomallei*-infected worms	Inhibition of transcription of the genes encoding virulence factors	[72]
Aloe-emodin rhein and emodin	Rhubarb	*S. aureus* ATCC 25923	Aloe-emodin = 7.5 mg/mL	All compounds rescued *C. elegans* infected with *S. aureus* 25,923 at concentration of <60 µg/mL, as detected using microfluidic device survival assay	N.D.**	[73]
			Rhein = 16.0 mg/mL			
			Emodin = 6.3 mg/mL			
Magnolol	*Scutellaria baicalensis,* *Scutellaria lateriflora,* *Magnolia officinalis*	*V. cholerae* O1 E1 Tor serotype Inaba strain N16961	Baicalein = 1.25 µg/mL	1–10 µg/mL Honokiol and Magnolol prolonged survival of *C. elegans* infected with *V. cholerae* O1	Biofilm inhibition, anti-inflammatory effects	[74]
Honokiol			Honokiol = 0.625 µg/mL			
Quercetin			Magnolol = 5 µg/mL			
Resveratrol			Quercetin = 0.625 µg/mL			
			Resveratrol = 0.625 µg/mL			

**Table 2:** Antifungal activity of natural plant products in the *C. elegans* in vivo model. * MoA: mechanism of action; ** N.D.: not determined.

Active Compounds	Plant	Microorganism Tested	In Vitro	In Vivo (*C. elegans* Infection Model)	MoA *	References
			MIC_50_	EC_50_		
Gallic acid and gallates (secondary metabolites)	Derived from various plants	*Candida* spp.	N.D. **	Increase nematodes’ lifespans at >1 µg/mL concentrations	N.D. **	[84]
		*Cryptococcus* spp.				
		*Paracoccidioides* spp.				
		*Histoplasma capsulatum*				
Enoxacin	*Tabebuia avellaneda*	*C. albicans*	Inhibit *C. albicans* biofilm formation in vitro	Prolong the survival of *C. albicans*-infected worms and prevent fungal–hyphal transition	N.D. **	[85]
Caffeic acid phenethyl ester (CAPE)						
Lapachol						
Magnolol and honokiol	*Magnolia officinalis*	*C. albicans*	Significantly suppress in vitro yeast cell adhesion, hyphal growth, and biofilm formation at 4–32 μg/mL concentrations	Prolongs the lifespan of *C. albicans*-infected worms	Downregulation of virulence genes (Ras1-cAMP-Efg1 pathway*)*	[86]
A2	Plant-derived natural products compound collection	*C. albicans*	27.5 µg/mL	55.1 µg/mL	Photosensitisation	[87]
A8			5.8 µg/mL	23.1 µg/mL		
A17			31.0 µg/mL	31.0 µg/mL		
A19			26.5.0 µg/mL	26.5 µg/mL		
A21			16.5 µg/mL	16.5 µg/mL		
A24			13.3 µg/mL	13.3 µg/mL		
A25 (saponins)			28.7 µg/mL	28.7 µg/mL		
				All compounds increase *C. albicans*-infected worms’ lifespans		
Tetrandrine	*Stephania tetrandra*	*C. albicans*	MIC = 16, 32, 64 mg/L	EC_50_ = 4, 8 16, 32 mg/L protects worms against *C. albicans* infections	QS and biofilm inhibition (Ras/cAMP pathway)	[88]

## Data Availability

Not applicable.
